# Characterization of cxorf21 Provides Molecular Insight Into Female-Bias Immune Response in SLE Pathogenesis

**DOI:** 10.3389/fimmu.2019.02160

**Published:** 2019-10-21

**Authors:** Valerie M. Harris, Kristi A. Koelsch, Biji T. Kurien, Isaac T. W. Harley, Jonathan D. Wren, John B. Harley, R. Hal Scofield

**Affiliations:** ^1^Arthritis and Clinical Immunology Program, Oklahoma Medical Research Foundation, Oklahoma City, OK, United States; ^2^Departments of Pathology and Medicine, College of Medicine, University of Oklahoma Health Sciences Center, Oklahoma City, OK, United States; ^3^Division of Rheumatology, School of Medicine, University of Colorado, Aurora, CO, United States; ^4^Department of Immunology and Microbiology, School of Medicine, University of Colorado, Aurora, CO, United States; ^5^Center for Autoimmune Genomics and Etiology, Cincinnati Children's Hospital Medical Center, Cincinnati, OH, United States; ^6^Department of Pediatrics, University of Cincinnati College of Medicine, Cincinnati, OH, United States; ^7^United States Department of Veterans Affairs Medical Center, Cincinnati, OH, United States; ^8^Medical and Research Services, Oklahoma City Department of Veterans Affairs Health Care Center, Oklahoma City, OK, United States

**Keywords:** sex-bias, systemic lupus erythematosus, innate immunity, X-chromosome, Interferon-inducible

## Abstract

**Background:** Ninety percent of systemic lupus erythematosus (SLE) patients are women. X chromosome-dosage increases susceptibility to SLE and primary Sjögren's syndrome (pSS). Chromosome X open reading frame 21 *(CXorf21)* escapes X-inactivation and is an SLE risk gene of previously unknown function. We undertook the present study to delineate the function of CXorf21 in the immune system as well as investigate a potential role in the sex bias of SLE and pSS.

**Methods:** Western blot protein analysis, qPCR, BioPlex cytokine immunoassay, pHrodo™ assays, as well as *in vitro* CRISPR-Cas9 knockdown experiments were employed to delineate the role of CXorf21 in relevant immunocytes.

**Results:** Expressed in monocytes and B cells, *CXorf21* basal Mrna, and protein expression levels are elevated in female primary monocytes, B cells, and EBV-transformed B cells compared to male cells. We also found *CXorf21* mRNA and protein expression is higher in both male and female cells from SLE patients compared to control subjects. TLR7 ligation increased CXorf21 protein expression and *CXorf21* knockdown abrogated TLR7-driven increased *IFNA1* mRNA expression, and reduced secretion of both TNF-alpha and IL-6 in healthy female monocytes. Similarly, we found increased pH in the lysosomes of *CXorf21*-deficient female monocytes.

**Conclusion:** CXorf21 is more highly expressed in female compared to male cells and is involved in a sexually dimorphic response to TLR7 activation. In addition, CXorf21 expression regulates lysosomal pH in a sexually dimorphic manner. Thus, sexually dimorphic expression of CXorf21 skews cellular immune responses in manner consistent with expected properties of a mediator of the X chromosome dose risk in SLE and pSS.

## Introduction

SLE is a chronic systemic autoimmune disease characterized by several simultaneous abnormalities, such as B cell hyperactivation, autoantibody production, and interferon signature. Moreover, while >90% of SLE patients are women, the pathophysiological mechanisms underlying this bias are incompletely defined. The etiology of SLE is attributed to both heritable and environmental factors (1, 2). Hormones, genetics, microbiota, infections, and lifestyle have all been hypothesized to have roles in the sex bias observed in SLE, however, the reports are conflicting and the cause remains ambiguous.

We have proposed an X-chromosome gene dose effect due to the difference in the number of X chromosomes between women (46,XX) and men (46,XY) as a mechanism for the female sex bias found in SLE and pSS, as well as for differences in overall immune responses (3–5). The imbalance in X-chromosome gene expression is equalized by random silencing of one X-chromosome. However, on the inactivated X-chromosome (X_i_), a number of genes escape inactivation giving women more variability in X-linked gene expression (6). Immune-related X-linked genes, such as TLR7, CD40L, DDX3X, and IL3RA, escape inactivation in female immune and somatic cell populations, as previously reported (7–10). Such disparity in immune-related protein-coding gene expression could provide an explanation for differences in male and female immune responses, as well as the susceptibility of women to autoimmune disease.

Chromosome X open reading frame 21 *(CXorf21)*, is an uncharacterized protein-coding gene that contains SLE risk allele, rs887369 (11). The X-linked CXorf21 gene has female-biased expression and in some cases has been shown to variably escape X-inactivation (9, 11, 12). *CXorf21* has been shown to variably escape X-inactivation and have female bias in expression in certain tissues including EBV-transformed B cells (GTEx Analysis Release V7 (dbGaP Accession phs000424.v7.p2) (6, 9, 12). *CXorf21* appears to be an interferon inducible gene as NF-kB, IRF1-3, and STAT1-3, among other transcription factors bind the *CXorf21* promoter region (13–15). Along with interferon regulatory genes, *CXorf21* mRNA expression levels in peripheral blood cells are indicators of SLE flares (16). Recently, Odhams et al. showed that CXorf21 expression is increased by LPS and IFN-gamma and IFN-alpha in monocytes and B cells (15). Additionally, this group found that CXorf21 co-localizes with lysosomal resident TLR7, and the *CXorf21* SLE risk allele (rs887369) increases expression. Taken together, these observations suggest that CXorf21 is a mediator of the X-chromosome gene dose-dependent increased risk of SLE in females.

Further evidence supporting a role for CXorf21 in innate immunity and in the pathogenesis of SLE and pSS come from The BioPlex Network, a publicly-available, unbiased protein-protein interaction screening project (17). This effort identified solute carrier family 15 (proton-oligopeptide co-transporter) member 4 (SLC15A4) (17) as a binding partner of CXorf21. Importantly, GWAS studies in both SLE (18) and pSS (19) have identified risk variants within *SLC15A4*. SLC15A4, a lysosome membrane protein, transports antigenic oligopeptide/amino acids and protons into the cytosol to initiate downstream cytosolic receptor signaling pathways. An *Slc15a4* loss-of-function mutation known as *feeble* is protective in an SLE-susceptible mouse model (20). SLC15A4 regulates lysosomal antigen processing, cytosolic NOD1-dependent NF-κB signaling, mTOR-dependent toll-like receptor (TLR) 7 cytokine secretion, and antibody production in various immune cell populations (20, 21).

These lysosomal processes are very pH sensitive. Consistent with a requirement for lysosomal acidification, *SLC15A4*-deficient mice are unable to transport basic amino acids/oligopeptides out of the lysosome, resulting in their accumulation, therein. It is predicted that the accumulation of basic amino acids/oligopeptides in the lysosome dysregulates the function of v-ATPase pumps and other proton transporters, which are required for the lysosome proton gradient (20). CXorf21, a direct protein-protein partner of SLC15A4, has a conserved short-chain dehydrogenase reductase domain, which may function as an NADP(H)-dependent oxidoreductase by utilizing hydrogen ions produced by SLC15A4 to generate NAPDH (dehydrogenase), or generate hydrogen ions (reductase) from NAPDH. Both processes are of interest, as lysosomal resident, NADPH oxidase 2 (NOX2), utilizes NAPDH to generate superoxides necessary for antigen processing and presentation (22). Thus, we predict that the expression of CXorf21 is important and possibly a rate-limiting step necessary for generating NAPDH for NOX2 function, or alternatively, it sequesters and reduces NAPDH, thus limiting NOX2 function, and generating protons required for other lysosomal proton-coupled transporters such as SLC15A4 and V-ATPase. Add to this, our previously published results showing that female monocytes, B cells, and dendritic cells, all which that overexpress CXorf21, had lower a lysosomal pH compared to male cells (23).

We undertook this study to investigate the influence of sex on the expression and function of CXorf21. We demonstrate herein that CXorf21 is required for SLC15A4-mediated TLR7 immune responses and the lysosomal pH gradient. Additionally, the female-biased expression of CXorf21 and TLR7 in these immune response pathways provide a mechanism through which the X-chromosome gene dosage effect contributes to the disparate risk of SLE between the sexes.

## Materials and Methods

### Patients/Donors

Whole blood was donated by volunteer healthy controls. Healthy female and male controls were recruited pair-wise to control for day-to-day variability. Lymphoblastoid cell lines (LCLs) derived from healthy controls and SLE patients with and without chromosomal aneuploidies were obtained from the Lupus Family Registry and Repository (24). All SLE subjects met the 1982 Revised Classification Criteria for SLE (25). All procedures were approved by Oklahoma Medical Research Foundation and University of Oklahoma Health Sciences Center IRBs. All subjects provided written informed consent.

### GAMMA

The phenotypic consequences of *CXORF21* disruption and potential genetic network for *CXORF21* (i.e., genes that potentially interact with or influence the expression) were predicted using previously developed software called GAMMA (Global Microarray Meta-Analysis) (26, 27). Briefly, GAMMA processes public transcriptional datasets within GEO (RNAseq and microarray) to identify genes that are highly correlated in their expression across experimental conditions. This “guilt by association” approach assumes that a gene's function and phenotype can be approximated by what its most highly correlated genes have in common, which is accomplished by literature-mining analysis (28). Since inception, this approach has been validated experimentally in a number of studies to date (29–38).

### Cell Culture

Primary monocytes were initially cultured in RPMI 1640 (Gibco) with no FBS and allowed to adhere to the culture plates overnight. Cells were washed with PBS to remove dead and non-monocyte cells. Media was replaced with Glutamax Opti-MEM I reduced serum medium (Thermofisher) supplemented with penicillin and streptomycin for transfection and activation experiments.

### Isolation of Cells

STEMCELL EasySep™ monocyte and B cell isolation kits were used to isolate monocytes and B cells, respectively, from the PBMCs of healthy control and SLE subjects. Briefly, PBMCs were first isolated by density gradient centrifugation using Lymphoprep (STEMCELL Technologies, Cambridge, MA) according to the manufacturer's protocol. Cells were resuspended in EasySep™ buffer, the EasySep™ Magnet was used to sequentially isolate CD14^+^ (using the EasySep™ Human CD14 enrichment kit) and CD19^+^ (using the EasySep Human CD19 positive selection kit II). Monocyte and B cell population purity was confirmed by Moxi-Flow cytometry with anti-CD14/CD16 (STEM CELL) and anti-CD45/CD19 (STEM CELL) markers with the protocol described here (39). Data were analyzed using FlowJo™.

### RNA Extraction and Quantitative Real-Time PCR (qPCR)

Trizol extractions were conducted according to the manufacturer's protocol (Invitrogen) as described (40). RNA concentrations were measured using Qubit (Invitrogen) according to the manufacturer's protocol. Complementary DNA (cDNA) was synthesized using the BioRad gDNA clear cDNA synthesis kit. qPCR experiments were prepared in 384-well plates using ABI7900 (Thermofisher). Sample master mixes were made with PrimePCR™ Taq probes [*CXorf21* (qHsaCIP0028546; intron-spanning probe targeting exon 1 and 2), *GAPDH* (qHsaCEP0041396), *TLR7* (qHsaCEP0055373**)**, or *IFNalpha1*(qHsaCEP0055508)],IFNbeta1 (qHsaCEP0054112), and PrimePCR™ Supermix.

### Western Blot Analysis

SDS-PAGE was carried out according to Laemmli et al. (41), except for using pre-cast 4–20% gradient gels (Bio-Rad). Gel proteins were transferred to nitrocellulose membranes using Trans-Blot Turbo transfer system and Trans-Blot Turbo transfer pack (Bio-Rad). Proteins were probed with anti-CXorf21 and anti-actin antibodies (Novus Biotechnologies) and detected with alkaline phosphatase/nitro blue tetrazolium/5-bromo-5-chloro-3-indolyl phosphate system. Protein bands were quantified using densitometry (ImageJ).

### CRISPR-Cas9 Transfection

Freshly isolated primary monocytes were seeded and allowed to adhere to 96-well plates (2 × 10^4^ cells per well), 24-well plates (1.2 × 10^5^ cells per well) or 6-well plates (5 × 10^5^ cells per well) in Opti-MEM I reduced serum medium supplemented with penicillin and streptomycin. Following cell adherence overnight, media were collected, non-adherent cells removed and media containing transfection reagents were added to the cells. *CXorf21* DNA primers (forward, TAATACGACTCACTATAGAACCAAGTAGGTCTCT, and reverse TTCTAGCTCTAAAACGCAGAGAGACCTACTTGGT) were designed using GeneArt™ CRISPR Search and Design Tool and *CXorf21* gRNA was synthesized and purified with GeneArt™ Precision gRNA synthesis kit according to manufacturer's protocol. The gRNA targets exon 1 of CXorf21 (chr.X:30560017-30560036) within the genomic DNA. Cas9 RNP nuclease and CXorf21 gRNA or kit-provided control gRNA complexes were formed according to CRISPRMAX Lipofectamine reagent protocol and cells were transfected for 48 h. Media were collected and replaced with fresh media after 8 h and allowed to incubate for an additional 40 h. At the end of the 48 h transfection period, media were changed again, and cells were allowed to recover overnight prior to TLR7 stimulation step. To confirm transfection efficiency, control wells were simultaneously transfected with GFP vector (Lonza). The adherent monocyte cells were stained with DAPI and accessed under the microscope for GFP 24 h post-transfection. Genomic Detection Cleavage kit (Thermofisher) was used according to protocol to determine the percentage of DNA that underwent Cas9 nuclease double cuts at the *CXorf21* locus. Gene Modification Efficiency (test sample) = 1– [(1-fraction cleaved) 1/2], with the fraction cleaved = sum of cleaved band intensities/(sum of the cleaved and parental band intensities).

### TLR7 and NOD1 Stimulation

Transfected and non-transfected primary monocytes or B cells were stimulated for 24 h with TLR7-agonist, R837 (1 μM), NOD1 agonist, ie-DAP (10 mM), or vehicle (endotoxin-free water (Miltenyi Bio).

### Cytokine Immunoassay

Cell culture media were collected pre-transfection (following overnight adherence) mid-transfection (8 h into the transfection period), and post-activation (24 h following TLR7-stimulation) of primary monocytes. Undiluted media samples were plated, and analytes (IL-1, IL-6, IL-8, IL-10, GM-CSF, and TNF-alpha) were detected according ProcartaPlex™ Platinum Human Multiplex Assays manufacturer's protocol. Plate was read on the BioPlex 200 (BioRad).

### Lysosomal pH Determination

To detect the intracellular pH in live primary monocytes, the pHrodolin®Red AM Intracellular pH Indicator kit (Molecular Probes) was used according to the manufacturer's suggested protocol, as we have reported (23). We determined the mean cellular fluorescence in triplicate samples using a spectrophotometer (Synergy H1, BioTek). A standard curve showed a linear relationship between the intracellular pH and the relative fluorescence units.

### Statistics

Statistical analyses were carried out using both parametric and non-parametric statistical test. Gaussian distribution was determined using D'Agostino-Pearson omnibus normality test. Student's *t*-test, Kruskal-Wallis one-way analysis of variance (ANOVA) with Dunn's multiple comparisons, or Fisher's Exact test were determined using GraphPad Prism 7 software.

## Results

### CXorf21 Is an Immune-Related Protein

**Figure 1:**
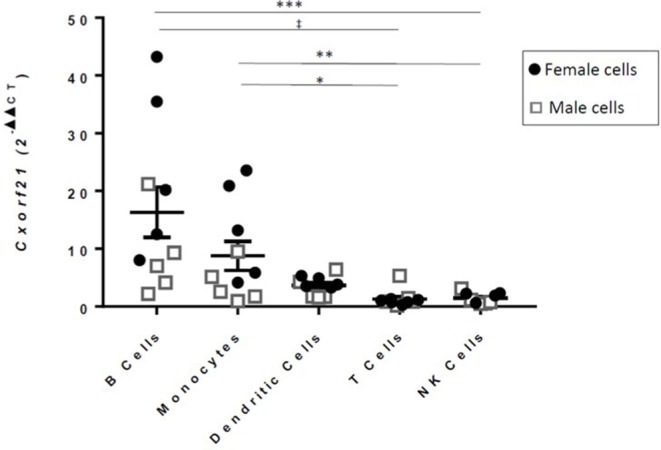
RT-qPCR of *CXorf21* from primary monocytes, dendritic cells, natural killer cells, B cells, and T cells of healthy male and female controls [12; females = 6 (black circles)/males = 6 (gray squares)]. One-way ANOVA Kruskal-Wallis non-parametric test with a Dunn's multiple comparisons. ^*^p < 0.05; ^**^p < 0.01; ^***^p < 0.001, ^‡^*p* < 0.0001. Error bars represent SEM.

CXorf21 protein function has yet to be determined. GAMMA predicted that CXorf21 is involved in immune function (Table 1, Table S1 for full table). This was supported by our quantitative real-time PCR data, which showed that the CXorf21 mRNA was highly expressed in antigen presenting cells, such as primary CD14^+^ monocytes, and CD19^+^ B cells (Figure 1). Coincidently, female monocytes and B cells (black circles) had the highest relative expression compared to male counterparts (gray squares) (Figure 1). Public protein (42) and RNA expression atlases (43, 44) as well as bioinformatic functional annotation prediction databases (45, 46) yielded similar results (Table S1 and Figure S1).

**Table 1:** Global meta-analysis (GAMMA^25^) scores predicted associations with *CXorf21* based compilation of publicly available microarray data and literature search.

**Predicted associations**	**GAMMA score**
B cell activation	115
Dendritic cells	102
B cells	85
Monocytes	55
TLR7	52
Cytokine production	45
Systemic lupus erythematosus	43
Antigen presentation	43
Type I IFN	43
IL-6	37
Proinflammatory cytokines	35

*A higher GAMMA score indicates a stronger predicted association. A complete table of predictions is shown in Table S1*.

**Figure 2:**
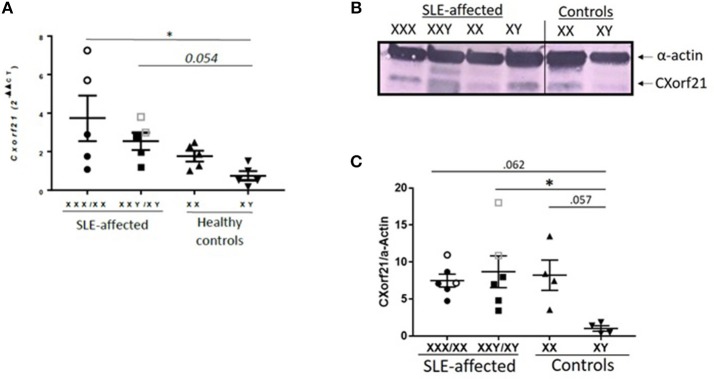
*CXorf21* is differentially expressed in primary immune cells of healthy controls and SLE-affected patients. **(A)** RT-qPCR of *CXorf21* mRNA expression in LCLs from SLE-affected females (*n* = 5) (47,XXX female patients are represented in clear circles), SLE-affected males (*n* = 5) (47,XXY male patients are represented in clear squares), healthy females (*n* = 5), and healthy males (*n* = 5). One-way ANOVA Kruskal-Wallis nonparametric test with a Dunn's multiple comparisons *p < 0.05; Error bars represent SEM. **(B)** Total protein extract was harvested from SLE-affected and unaffected male and female LCLs were subjected to SDS-PAGE. Western blotting using human anti-CXorf21 antibody (34 kD) identifying bands at the appropriate molecular weight. Human anti-alpha actin (42 kD) is shown as a loading control. (l-r) Lane 1: 47,XXX female lupus patient, lane 2: 47,XXY male lupus patient, lane 3: 46,XX female lupus patient, lane 4: 46,XY male lupus patient, lane 5: 46,XX female control patient, lane 6: 46,XY male control patient. This blot is representative of 4 different experiments and the order was rearranged to correlate with mRNA graph in this figure. **(C)** Quantitative protein analysis of CXorf21/Actin was determined for all blots using GelAnalyzer (47,XXX and 47,XXY patients are represented in clear circles and squares). A nonparametric ANOVA was performed using to determine statistical significance.

CXorf21 has been shown to escape X-inactivation and have female biased expression in various tissues and whole blood (6, 9, 12, 15) by quantitative measurement of mRNA. We have previously shown that CXorf21 protein was increased in healthy female monocytes, B cells, and LCLs compared to healthy male samples (23), therefore, we assessed whether SLE-affected and unaffected subjects, with and without X-chromosomal aneuploidies, have increased expression of CXorf21 (Figures 2A,B). We have previously reported that an X-chromosome gene dose effect underlies the female bias of these diseases (3–5). In LCLs derived from SLE-affected subjects, we found that expression levels of CXorf21 were increased in 47,XXX/46,XX women (3.8-fold), 47,XXY/46,XY men (2.3-fold) compared to healthy male control LCLs (Figure 2A). Protein analysis confirmed that CXorf21 levels increased in 46XY and 47,XXY men with SLE (Figures 2B,C). Initial analysis of non-B/non-T PBMCs from patients with primary Sjogren's syndrome, another autoimmune disorder with a female to male bias of >13:1 (47), showed an increase (2.3-fold) in CXorf21 expression similar in degree to that found in SLE, but not statistically significant compared to healthy male control samples (data not shown).

### Knockdown of CXorf21 Modulates NOD1-Mediated SLC15A4 Function and Lysosomal pH in Female Monocytes

**Figure 3:**
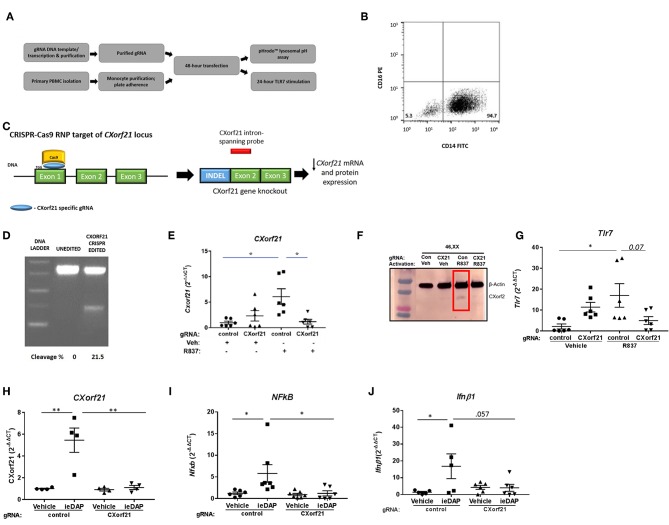
CRISPR-Cas9 knockdown of *CXorf21* mRNA and protein silences CXorf21. **(A)** Workflow of experiment. **(B)** Purification of classical CD14+/CD16- monocytes population were confirmed using flow cytometry. **(C)** Cas9 nuclease RNP targeting exon 1 of *CXorf21* locus subsequently resulted in decreased detection of *CXorf21* transcript by intron-spanning *taq* probe. **(D)** Cas9-gRNA efficiency (18%) was detected enzymatically by GeneArt™ Genomic Detection Kit. Percentage of cleavages was determined with Gel Analyzer and calculation described in methods. **(E)** RT-qPCR of *CXorf21* mRNA expression from primary monocytes from 46, XX female transfected with control (Con) or *CXorf21*-specific gRNA (CX21). Ligand stimulation were performed for 24 h with vehicle (Veh) or R837 (1 μM). Fold changes for female samples show relative expression to veh-treated control gRNA female monocytes. Data represents monocytes from 6 female subjects plated in replicates of 3. Error bars represent SEM. One-way ANOVA Kruskal-Wallis nonparametric test with a Dunn's multiple comparisons. *p < 0.05. **(F)** Total protein extract from 46,XX female primary monocytes subjected to SDS-PAGE. Western blotting using human anti-CXorf21 antibody (34 kD) identifying bands at the appropriate molecular weight. Human anti-alpha actin (42 kD) is shown as a loading control. (l-r) lane 1 (control gRNA) and 2 (*CXorf21* gRNA) are healthy 46,XX female monocytes treated with vehicle and lanes 3 and 4 are treated with R837**. (G)** RT-qPCR of *Tlr7* mRNA expression from female primary monocytes transfected with control (Con) or CXorf21-specific gRNA (CX21). Ligand stimulation were performed for 24 h with vehicle (Veh) or R837 (1 μM). Fold changes for female samples show relative expression to female Con-Veh. RT-qPCR of **(H)** *CXorf21*, **(I)** *NFkB1*, and **(J)** *Ifnb1* mRNA expression from primary monocytes from 46, XX female transfected with control (Con) or *CXorf21*-specific gRNA (CX21). Ligand stimulation was performed for 24 h with vehicle (Veh) or NOD1 agonist ie-DAP (10 mM). **p < 0.01.

While the function of CXorf21 is still unknown, published data show that CXorf21 interacts with SLC15A4 (17), and *Slc15a4*-deficient cells had decreased TLR7-stimulated immune response and increased lysosomal pH (20). Thus, we predict that knock down of CXorf21 expression in primary monocytes may recapitulate what was observed with manipulation of *Slc15a4* expression. Initial knockdown experiments were performed with siRNA-lipofectamine transfection protocols, however low transfection efficiency and activation of the TLR7 pathway with the siRNA was problematic. Consequently, our experimental design was modified to use CRISPR-Cas9 RNPs/CXorf21-targeted small guide RNAs in primary human monocytes (Figure 3A). We isolated primary classical CD14^++^/CD16^−^ monocytes from healthy control females (Figure 3B) and allowed them to adhere to culture plates to further purify the cell population. Small guide RNA (gRNA) targeting exon1 of the *CXorf21* locus and Cas9 RNPs were used to induce indels, and an intron-spanning *CXorf21* probe was used to identify the presence of *CXorf21* transcripts (Figure 3C). This CXorf21-targeted gRNA-Cas9 complex resulted in 21.5% indels (Figure 3D). We observed successful attenuation of TLR7-stimulated increase in *CXorf21* mRNA (Figure 3E) and protein (Figure 3F) in the female monocytes after inhibition of CXorf21. Expression of *CXorf21* mRNA (Figure 1) and protein (23) in male monocytes ranged from low to undetectable, hence there was no change in relative expression of CXorf21 following knockdown and subsequent stimulation in male primary monocytes (Figure S2A). Interestingly, we did find that knockdown of CXorf21 decreased R837-activated *TLR7* mRNA expression in primary female monocytes, suggesting a feedforward interaction between the two proteins (Figure 3G). As an additional control, we tested SLC15A4 function following CXorf21 knockdown by activating female monocytes with a NOD1 agonist, i.e., DAP. NOD1 antigens are transported into the cytosol by SLC15A4 and result in downstream NF-kappaB signaling and cytokine production (48). We show that NOD1 activation following knockdown of CXorf21 in female monocytes (Figure 3H), resulted in decreased NOD1-mediated *Nfkb1* (Figure 3I) and *Ifnb1* (Figure 3J) expression. These data demonstrate that knockdown of CXorf21 in primary monocytes disrupts SLC15A4 function, and the regulation of *Tlr7* expression.

**Figure 4:**
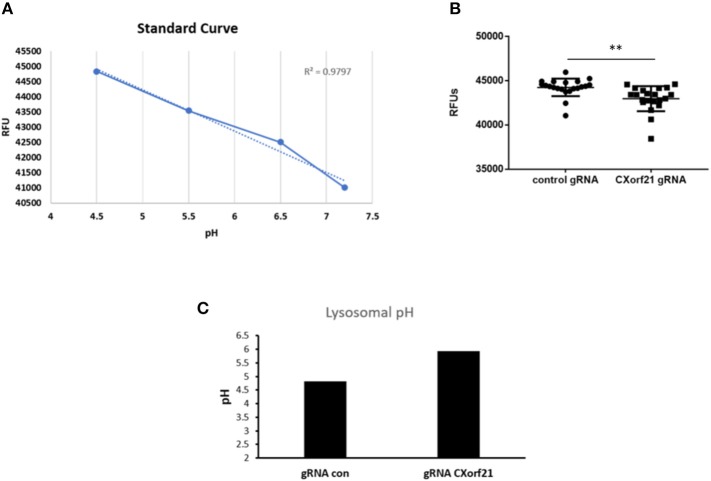
Knockdown of CXorf21 increases lysosomal pH in 46,XX female monocytes. **(A)** Standard curve using pHrodo™ Red AM with Intracellular pH Calibration Buffer kit for the translation of fluorescence ratios in pH. An average of six data points was plotted in the graph and a linear trend line was fit to get a pH standard curve. Female monocytes transfected with control or *CXorf21* gRNA were stained with pHrodo™ Red solution and **(B)** relative fluorescence units (RFUs) were measured with multi-well plate reader (details in section Materials and Methods). *N* = 8; error bars represent SEM; **p = 0.001. **(C)** pH was determined based on RFUs using the standard curve.

With inhibition of CXorf21 expression, we found altered lysosomal pH in female monocytes. Knockdown of CXorf21 increased the pH from 4.6 to 5.8 (Figure 4), thus altering the lysosomal environment. This is further evidence of the importance of female overexpression of X-linked immune proteins and the resulting disparate immune response observed in women.

### Knockdown of CXorf21 Regulates IL-6, TNF-Alpha, and IFN-Alpha in Female Monocytes

**Figure 5:**
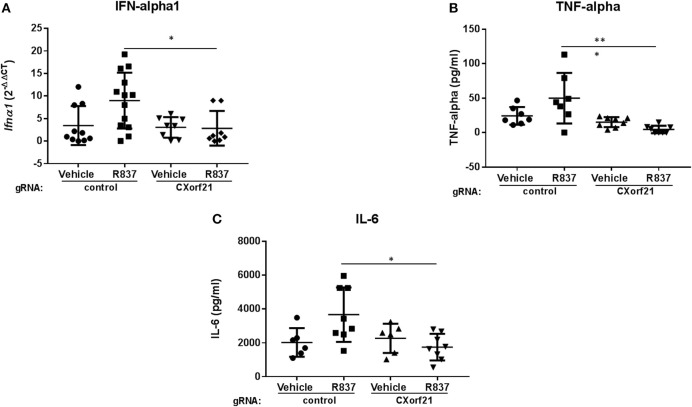
Knockdown of *CXorf21* decreases TLR7-stimulated IFN-alpha1, TNF-alpha, and IL-6. **(A)** RT-qPCR of Ifna1 mRNA expression from 46, XX female primary monocytes transfected with control (Con) or *CXorf21*-specific gRNA (CX21). Ligand stimulations were performed for 24 h with vehicle (Veh) or R837 (1 μM). Fold changes show relative expression compared to female Con-Veh. **(B)** TNF-alpha and **(C)** IL-6 plots show cytokine production in 46, XX female primary monocytes transfected with control (Con) or CXorf21-specific gRNA (CX21). Ligand stimulation was performed for 24 h with vehicle (Veh) or R837 (1 μM) and cytokine in media detected via ProcartaPlex™ Platinum Human Multiplex Assays. Error bars represent SEM. *p > 0.05, ***p < 0.001.

Next, we tested whether CXorf21 is critical for cytokine production. While adherence to the plastic plate did not cause increased levels of cytokine production from the primary monocytes, we did expect that CAS9 RNP-gRNA would activate the cells, and it did. For this reason, 8 h following transfection we replaced the CAS9/gRNA-containing media with fresh media to remove residual transfection agents. Additionally, at the end of the 48 h transfection period, we replaced the media again, and allowed the cells to recover overnight. We found that, following CXorf21 knockdown, female monocytes treated with a TLR7 agonist had a 7-fold decrease in *IFNA1* expression compared to vehicle-treated cells transfected with control gRNA and knockdown of CXorf21 diminished this effect (Figure 5A). We also found that TNF-alpha (Figure 5B) and IL-6 (Figure 5C), but not IL-8, IL1, IL-10 or GM-CSF (Figure S3), were regulated by CXorf21 expression levels in female monocytes.

**Figure 6:**
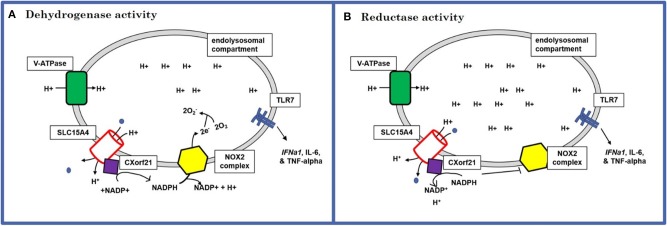
Schematic of the predicted mechanism of action for CXorf21. **(A)** Dehydrogenase activity: use protons provided by SLC15A4 along with NAPD^+^ to produce NADPH. NADPH can, in turn, be used by NOX2 complex to generate lysosome superoxide necessary for antigen processing (increases lysosomal pH). **(B)** Reductase activity: sequesters NAPDH to generate NAPD^+^ and protons. This could result in increased available hydrogen ions for proton transporters and pump, as well as limit NOX2 ability to produce superoxide anions (decrease lysosomal pH).

These data suggest that CXorf21 is important for specific cytokine production via the TLR7 signaling pathway and this response is female biased. As a working model we propose that putative SLE risk genes TLR7, CXorf21, SLC15A4 work together to regulate specific immune responses and lysosomal pH in female monocytes (Figures 6A,B). Specifically, based on published models of endolysosome role in inflammatory responses (49), we predict CXorf21 interacts with SLC15A4 to aid in modulation of NAPDH or H+ production, which may directly or indirectly affect the function of other endolysosomal proteins (i.e., TLR7, NOX2, V-ATPase pumps, other proton pumps, etc.) and their downstream responses (i.e., cytokine production, lysosomal pH).

## Discussion

One potential explanation for the gender bias in SLE is sexually dimorphic expression of genes escaping X-inactivation in men with X-chromosomal aneuploidies or women with two or more X-chromosomes. We have previously demonstrated an increased prevalence of Klinefelter's syndrome (47,XXY) and trisomy X (47,XXX), among men and women, respectively, with SLE or pSS (3–5). We propose the dimorphic overexpression of X-linked proteins, such as CXorf21 and TLR7, which the present work shows interact functionally in innate immune responses, contribute to the pathogenesis of female-biased autoimmune disorders such as SLE and pSS, and may explain, in part, the propensity for women to have these diseases.

GAMMA software results, along with our data demonstrating CXorf21 is expressed in CD33^+^ lineage cells (monocytes, dendritic cells, macrophages) and B lymphocytes, suggested an immunological role for CXorf21. We observed that CXorf21 was expressed in a female-biased manner in primary monocytes, primary B cells, and LCLs. Others have reported that TLR7 is biallelically expressed in female and Klinefelter's syndrome male primary monocytes and B cells (8) and our data confirmed that TLR7 was increased in male vs. female monocytes similar to CXorf21. These data, along with published data showing that the CXorf21 interacting partner, SLC15A4, regulates TLR7 downstream signaling (20) led us to examine whether knockdown of *CXorf21* would also abolish TLR7 immune response. Our data demonstrated that reducing expression of *CXorf21* in female primary monocytes resulted in decreased IL-6 and TNF-alpha cytokine production, but not other cytokines. There was also a marked decrease in *IFN-alpha1* mRNA expression following *CXorf21* knockdown. Thus, CXorf21 is required for specific TLR7 downstream immune responses in female primary monocytes. These results are complementary to recent data published by Odhams et al. that show *CXorf21* expression is LPS (TLR4) and interferon inducible (IFN-α and β) in monocytes in B cells and colocalizes with endolysosomal resident TLR7 (15). This study shows that both *CXorf21* mRNA and protein are more highly expressed in female vs. male monocytes. Additionally, this is the first study to show that TLR7 activation amplifies this response in women as compared to men.

An important component of the innate immune response is endolysosomal antigen processing. Subtle changes in lysosomal pH may result in disruption of antigen processing and cross-presentation, as well downstream immune responses (50, 51). We performed studies measuring changes in lysosomal pH following CXorf21 knockdown, and showed a less acidic lysosomal pH. Impaired lysosomal acidification has been observed in *Slc15a4*-/- mice and suggested that loss of the proton/oligopeptide co-transporter dysregulates the v-ATPase pump proton gradient caused by accumulation of histidine-containing amino acids and loss of protons entering the lysosome (20, 21). APCs such as DCs and B cells are more susceptible to subtle changes in pH; thus, altering their proteolytic activity (51, 52). The present study gives evidence that loss of CXorf21 in TLR7 activated cells leads to the same functional consequences in monocytes, such as change in cytokine production and lysosomal pH environment. We predict that knockdown of CXorf21 blocks SLC15A4 function, thus, increasing basic oligopeptides in the lysosome, which in turn makes the environment more alkaline. Additionally, loss of CXorf21 could result in diminished NADPH breakdown, resulting in reduced NOX2 activity, causing increased lysosomal superoxide production and increased lysosomal pH. Subsequently, the buffering capacity of these molecules would alter the proton gradient required for v-ATPase proton pump and other lysosomal proton channels to properly function. We have recently reported (23) that female cells that overexpress CXorf21 had a lower lysosomal pH compared to male samples, thus providing further support for CXorf21 in the role of lysosomal pH environment maintenance.

We propose that X-linked *CXorf21*, and *TLR7*, both putative risk genes in SLE that escape X-chromosome silencing, work together to drive the innate immune response in women, in a manner distinct from that found in men (53, 54). This provides a mechanism for the observed X-chromosome gene dose effect in the female-biased disorders, SLE, and pSS (3–5). These data show that SLE patients have increased CXorf21 expression compared to healthy control males. Another group also showed that having the CXorf21 risk haplotype resulted in increased expression of CXorf21(15). Interestingly, the CXorf21 expression in LCLs from SLE-affected Klinefelter's syndrome and 46,XY men mirrored and/or surpassed both SLE-affected, healthy control female or male expression. A limitation of our study was that the number of SLE and pSS patients and matched controls studied were small, as the number of available subjects visiting our clinic and available for research was limited. Future studies will include additional SLE and control sample that will allow us to increase our n and also study various immune cell types. It was encouraging that while the indel efficiency using Cas9 nucleases was not high, we still observed significant differences in function with knockdown. However, the more than 50% inhibition with only 21% indels suggest there may be other factors or off target effects that may be driving further repression of CXorf21. Nuclease-deficient Cas9 nucleases (dCas9) (55), which lacks endonucleolytic activity and specifically represses target gene transcription, may provide more complete knockdown and will be utilized in our future studies. Additionally, we have yet to study several other immune-related X-chromosome genes that may also contribute the X-chromosome gene dose effect.

Our previous results concerning X-chromosome aneuploidies demonstrated an increased prevalence of Klinefelter's syndrome and trisomy X in SLE and pSS but not in rheumatoid arthritis or primary biliary cholangitis, both of which have strong female bias (3–5). Thus, there is likely more than one pathway to female bias in autoimmune disease. Increased expression of interferon-regulated genes in peripheral blood cells in these diseases with an X chromosome dose effect (56–58), the so-called interferon signature, is a manifestation of pathogenesis. Our data show CXorf21 is involved in TLR7 signaling and IFN production and has a differential role between the sexes. Thus, we propose that the mechanism we have identified involving increased expression of CXorf21 in female immune cells that affects TLR7 signaling and interferon will be operative in autoimmune diseases in which the pathogenesis involves TLR7 signaling and an interferon signature. That there is more than one pathway to sex bias in autoimmune disease may be true even within a single disease entity, such as SLE, in that interferon production mediated through TLR7 signaling, which is initiated by binding of RNA by TLR7 in the lysosomal compartment, is only found in about 40% these patients.

This study provides insight into the functional immune roles for the uncharacterized and unsilenced CXorf21 protein and, moreover, is an integral piece into understanding the role this gene, in collaboration with other SLE putative risk genes, *SLC14A4*, X-linked *TLR7*, and potentially *NCF1* (NOX2 subunit) contribute to the X-chromosome dose effect that explains the female bias in SLE and pSS.

## Data Availability

All datasets generated for this study are included in the article/Supplementary Material.

## Ethics Statement

The studies involving human participants were reviewed and approved by OUHSC IRB committee. The patients/participants provided their written informed consent to participate in this study.

## Author Contributions

VH, BK, IH, KK, JW, JH, and RS contributed to the conception or design of the work, the acquisition, analysis, and interpretation of data for the work; drafted the work or revised it critically for important intellectual content; provided approval for publication of the content; agreed to be accountable for all aspects of the work in ensuring that questions related to the accuracy or integrity of any part of the work are appropriately investigated and resolved.

### Conflict of Interest Statement

The authors declare that the research was conducted in the absence of any commercial or financial relationships that could be construed as a potential conflict of interest.
