# Supplementary material for: Characterization of cxorf21 Provides Molecular Insight Into Female-Bias Immune Response in SLE Pathogenesis
Source: Front Immunol. 2019 Oct 21;10:2160. doi: 10.3389/fimmu.2019.02160 (PMC6816314; doi:10.3389/fimmu.2019.02160)
Supplement: Supplementary file 1 [file Data_Sheet_1.pdf]

Supplementary Figures

Table S1. Full table of Global meta-analysis (GAMMA[25]) scores predicted associations with CXorf21 based compilation of publicly available microarray data

Predicted association	Scores
**IRF4**	117
**B cell activation**	115
**B cell antigen receptor**	108
**dendritic cell**	102
**VAV1**	97
**CD32**	88
**B Cells**	85
**CD38**	73
**DAP12**	73
**T cell activation**	71
**IL-4**	71
**MHC class II**	69
**T Cells**	66
**SH2D1A**	66
**chronic lymphocytic leukemia**	65
**CD22**	64
**CD86**	63
**CARMA1**	63
**CD11B**	61
**ZAP-70**	61
**RAC2**	58
**Monocytes**	55
**tyrosine phosphorylation**	55
**T cell development**	55

**linker for activation of T cells**	55
**SYK**	54
**TLR2**	54
**Macrophages**	53
**CD19**	53
**CD80**	52
**TLR7**	52
**AML**	51
**IFN-gamma**	50
**CR2**	50
**innate immunity**	49
**CCR7**	49
**LYN**	49
**class switch recombination**	49
**B-cell lymphoma**	48
**signal transduction**	47
**pleckstrin**	47
**p65**	47
**B cell development**	47
**B-CLL**	46
**OCA-B**	46
**cytokine production**	45
**cell surface receptors**	45
**LFA-1**	45
**ERK1**	44
**F-actin**	44
**NK cells**	43
**systemic lupus erythematosus**	43

**T cell proliferation**	43
**antigen presentation**	43
**type I IFN**	43
**B cell proliferation**	43
**collagen receptor**	42
**Toll-Like Receptors**	41
**CD69**	41
**HLA-DRA**	40
**Bcr-Abl**	39
**SH3 domain**	39
**Interferon**	38
**TLR4**	38
**hematopoietic stem cell**	38
**C-Kit**	38
**cell surface**	37
**rheumatoid arthritis**	37
**IL-6**	37
**SLP-76**	37
**immunological synapse**	37
**NF-kappaB**	36
**CD28**	36
**p38 MAPK**	36
**BCL6**	36
**signaling lymphocytic activation molecule**	36
**lipid rafts**	36
**lymphocyte activation**	36
**T cell antigen**	36

**proinflammatory cytokines**	35
**tyrosine kinase**	35
**MHC class I**	35
**MHC**	35
**CD8+ T cells**	35
**CD45**	35
**protein kinase C**	35
**GPVI**	35
**B29**	35
**immunoreceptor**	34
**cytoplasmic domain**	34
**Granulocytes**	34
**IL-2**	34
**JNK**	34
**STAT3**	34
**CD14**	34
**STAT1**	34
**IL-2 receptor**	34
**A20**	34
**inflammatory response**	33
**tyrosine phosphatase**	33
**CD40L**	33
**antigen receptor**	33
**PU.1**	32
**autoimmune diseases**	32
**mitogen-activated protein kinase**	32
**wiskott-Aldrich syndrome protein**	32
**cd4+ T cell**	31

**CXCR4**	31
**Chemokine Receptor**	31
**actin polymerization**	31
**B cell differentiation**	31
**IL-10**	30
**IL-1beta**	30
**Chemokine**	30
**SHP-1**	30
**cell motility**	30
**plasma cell differentiation**	30
**immune response**	29
**CD4**	29
**chemotaxis**	29
**GM-CSF**	29
**PKB**	29
**IFN-gamma production**	28
**IFN-ALPHA**	28
**heterodimer**	28
**Melanoma**	28
**CD2**	28
**GEF**	28
**CD16**	27
**Integrin**	27
**Leukemia**	27
**hematopoiesis**	27
**ERK2**	27
**COLORECTAL CANCER**	27
**IL-3**	27

**memory B cells**	27
**M-CSF**	26
**C-FMS**	26
**Phosphatidylinositol 3-Kinase**	26
**c-Jun**	26
**protein tyrosine phosphatase**	26
**activated macrophages**	26
**CD18**	26
**CD44**	26
**kinase activity**	26
**C-CBL**	26
**IL-8**	25
**phagocytosis**	24
**adapter protein**	24
**NK receptors**	24
**Src family kinases**	24
**RANTES**	24
**CDC42**	24
**cell migration**	24
**G protein-coupled receptors**	24
**actin cytoskeleton**	24
**multiple sclerosis**	23
**Thymocytes**	23
**adhesion molecules**	23
**Normal B cells**	23
**Tumor suppressor**	23
**oncogene**	23
**NF-kappaB activation**	22

**DNA methylation**	22
**eosinophils**	21
**homing**	21
**ICAM-1**	21
**PTEN**	21
**extracellular signal-regulated**	20
**Caspase**	20
**Colitis**	20
**NSCLC**	20
**receptor tyrosine kinase**	19
**IL-12**	19
**CD49D**	19
**cytokine secretion**	18
**IL-18**	18
**STAT5**	18
**dephosphorylation**	17
**leukocyte antigen**	17
**RHOA**	17
**cell maturation**	17
**CD63**	17
**PKC-delta**	17
**RANKL**	16
**Zymosan**	16
**CHRONIC MYELOID LEUKEMIA**	16
**Hypermethylation**	16
**TP53**	16
**activator of transcription**	16
**neuroinflammation**	16

**promyelocytic leukemia**	16
**JAK1**	16
**peritoneal macrophages**	15
**NADPH Oxidase**	15
**beta-catenin**	15
**SCID**	15
**IL-17**	15
**alternatively spliced**	14
**ubiquitination**	14
**MULTIPLE MYELOMA**	12
**Caspase-8**	12
**regulatory T cells**	11
**inhibition of apoptosis**	11
**MAPK signaling**	10
**CREB**	10
**transcription start site**	10

TABLE S1. CXorf21 is an immune related protein. Complete table of Global meta-analysis (GAMMA[25]) scores predicted associations with CXorf21 based compilation of publicly available microarray data and literature search

Figure S1. http://ds.biogps.org/?dataset=GSE1133&gene=80231 BIOGPS: Expression of CXorf21 in human primary cells. This meta-analysis of 745 human primary cell samples shows changes in expression levels of CXorf21 (probe:220252_x_at) in both immune and non-immune related samples. Original source of these meta-data, Mabbot et al [29,30] combined over 100 publicly available microarray datasets derived from human primary cells.

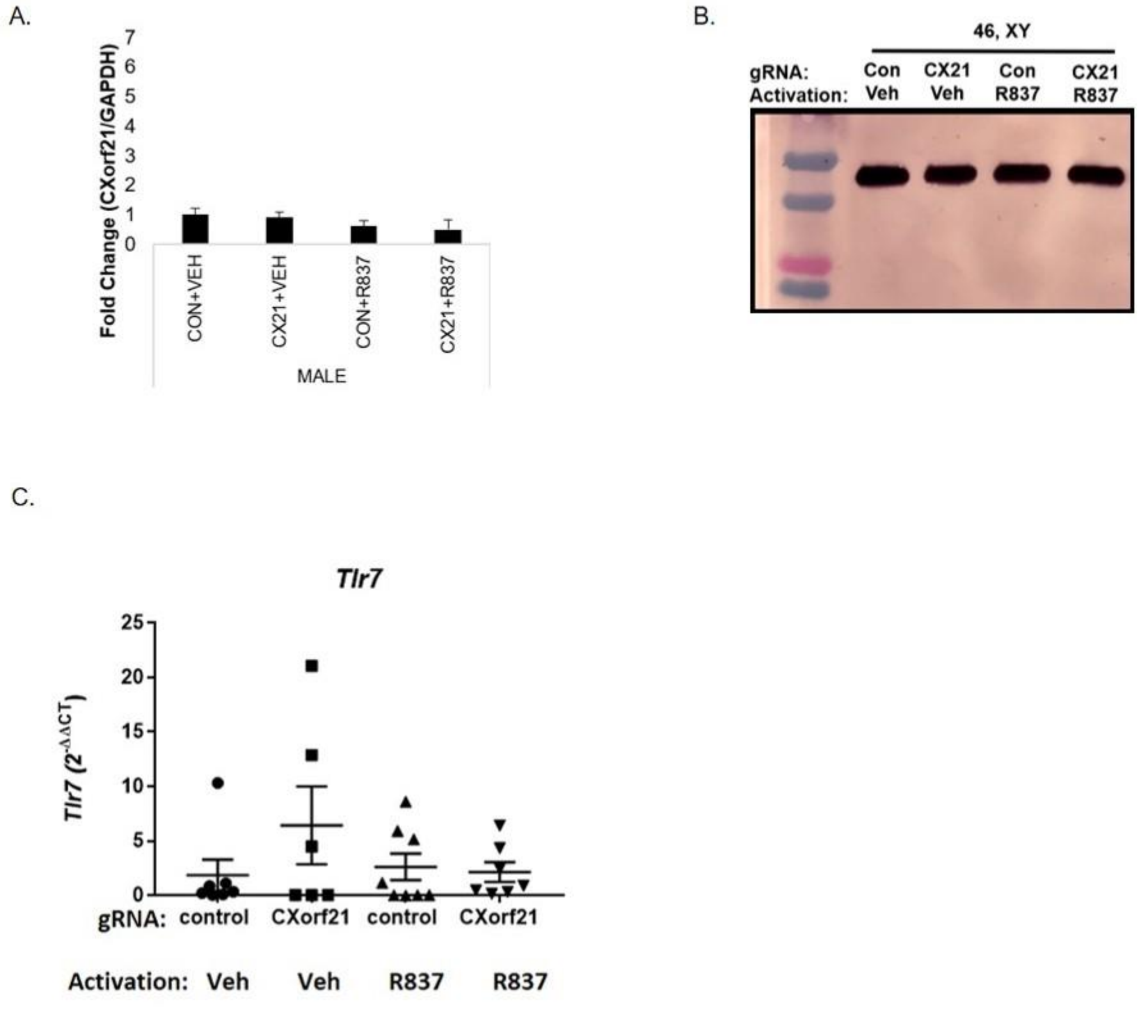

Figure S2. CRISPR-Cas9 knockdown of CXorf21 has no effect on *CXorf21* and*Tlr7* expression in male monocytes. (A.) RT-qPCR of *CXorf21* mRNA expression from primary monocytes from either healthy 46,XY males with control (Con) or *CXorf21*-specific gRNA (CX21). Ligand stimulation were performed for 24 hours with vehicle (Veh) or R837 (1μM). Fold changes for male samples show relative expression to male Con-Veh. Data represents monocytes 6 male in replicates of 3. Error bars represent SEM. Data was not significant. B. Total protein extract from 46,XY male primary monocytes subjected to SDS-PAGE. Western blotting using human anti-CXorf21 antibody (34kD) identifying bands at the appropriate molecular weight. Human anti-alpha actin (42kD) is shown as a loading control. (l-r) lane 1 and 2 is healthy 46,XY male monocytes treated with vehicle following transfection with control (Con) or *CXorf21*-specific gRNA (CX21), R837. Lane 3 and 4 samples were treated with R837 following transfection. C. RT-qPCR of TLR7 mRNA expression from male primary monocytes transfected with control (Con) or CXorf21-specific gRNA (CX21). Error bars represent SEM. One-way ANOVA Kruskal-Wallis nonparametric test with a Dunn's multiple comparisons. *p-values* *<0.05.

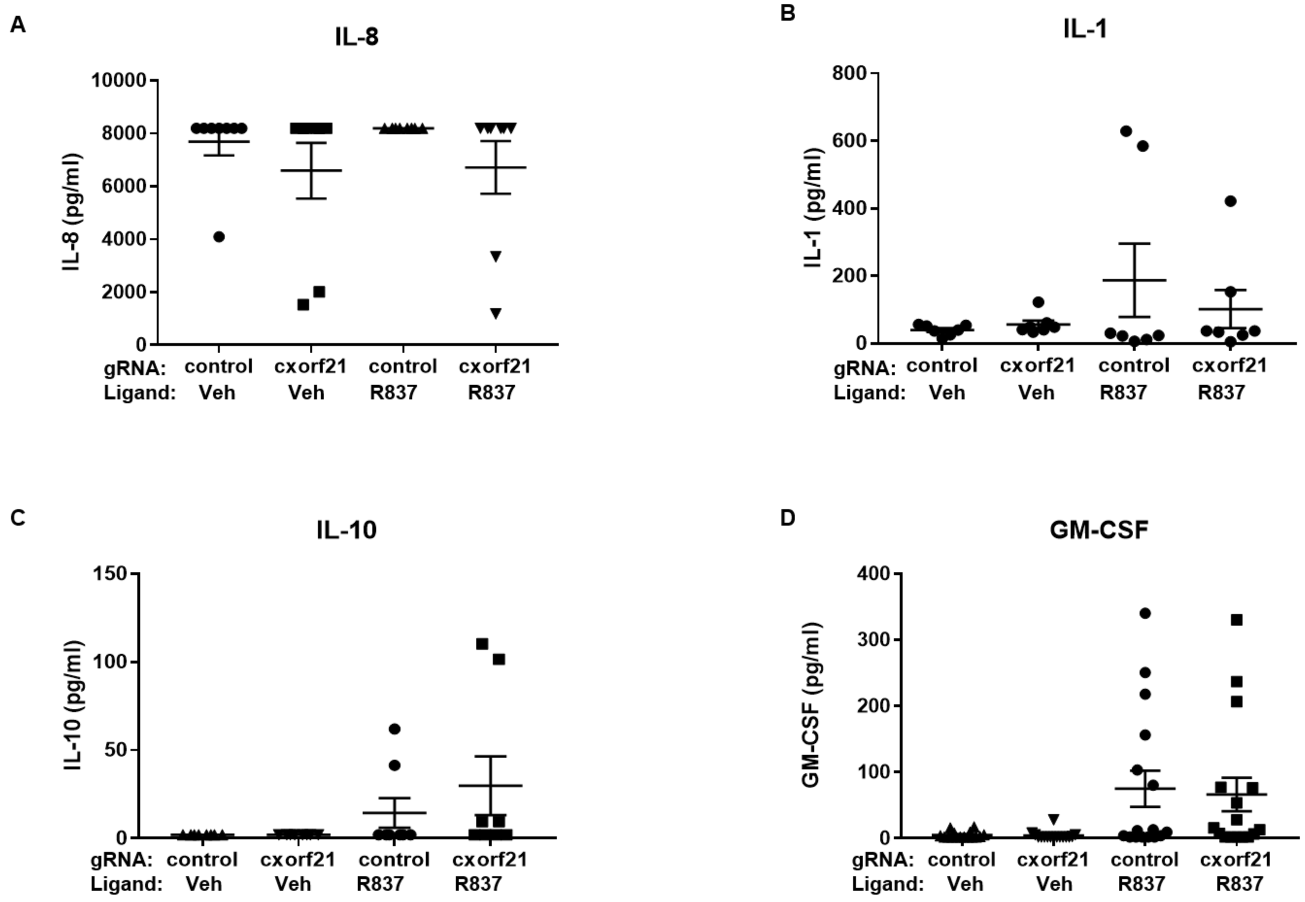

Figure S3. Regulation of inflammatory cytokines following *CXorf21* knockdown in primary monocytes. (A.) IL-8, (B.) IL-1, (C.) IL-10 and (D.) GM-CSF plots show cytokine production in 46, XX female primary monocytes transfected with control (Con) or CXorf21-specific gRNA (CX21). Ligand stimulation were performed for 24 hours with vehicle (Veh) or R837 (1μM) and cytokine in media detected via ProcartaPlex™ Platinum Human Multiplex Assays. Error bars represent SEM.
